# The effect of the COVID-19 pandemic on UK parent experiences of pregnancy ultrasound scans and parent-fetal bonding: A mixed methods analysis

**DOI:** 10.1371/journal.pone.0286578

**Published:** 2023-06-02

**Authors:** Emily Skelton, Alison Smith, Gill Harrison, Mary Rutherford, Susan Ayers, Christina Malamateniou

**Affiliations:** 1 Division of Radiography and Midwifery, School of Health and Psychological Sciences, City, University of London, London, United Kingdom; 2 Guy’s & St Thomas’ NHS Foundation Trust, London, United Kingdom; 3 Society and College of Radiographers, London, United Kingdom; 4 Perinatal Imaging and Health, King’s College London, London, United Kingdom; 5 Centre for Maternal and Child Health Research, School of Health and Psychological Sciences, City, University of London, London, United Kingdom; University of Mississippi Medical Center, UNITED STATES

## Abstract

**Introduction:**

Companionship in antenatal care is important for facilitating positive parental experiences. During the COVID-19 pandemic, restrictions on partner attendance at fetal ultrasound scans were introduced nationally to minimise transmission of the virus. This study aimed to explore the effect of these restrictions on maternal and paternal experiences of pregnancy scans and evaluate their potential effect on parent-fetal bonding.

**Methods:**

A UK-wide, anonymous cross-sectional survey was completed by new and expectant parents (n = 714) who had, or were awaiting a pregnancy scan during the COVID-19 pandemic. The CORE-10 and an adapted version of the Prenatal Attachment Inventory were used to evaluate psychological distress and prenatal bonding. Additional survey questions captured parental experiences of scans. Separate statistical and thematic analyses of the data were undertaken. A joint display matrix was used to facilitate integration of quantitative and qualitative claims to generate a comprehensive interpretation of study findings.

**Findings:**

When fathers did not attend the scan, feelings of excitement and satisfaction were significantly reduced (p<0.001) and feelings of anxiety increased (p<0.001) in both parents. Mothers were concerned about receiving unexpected news alone and fathers felt excluded from the scan. Mean paternal bonding (38.22, SD 10.73) was significantly lower compared to mothers (47.01, SD 7.67) although no difference was demonstrated between those who had attended the scan and those who had not. CORE-10 scores suggested low-to-mild levels of psychological distress, although the mean difference between mothers and fathers was not significant. Key themes described both parents’ sense of loss for their desired pregnancy scan experience and reflected on sonographers’ central role in providing parent-centred care during scans.

**Conclusion:**

Restrictions on partner attendance at scans during the COVID-19 pandemic had a negative effect on parental experiences of antenatal imaging. Provision of parent-centred care, which is inclusive of partners, is essential for improved parental experiences.

## Introduction

During the COVID-19 pandemic, significant changes were made to the provision of antenatal and intrapartum care to incorporate guidance for physical distancing and minimise virus transmission [1]. These recommendations prioritised the safety of the general population and healthcare staff, and aimed to reduce their risk of contracting the virus [2]. In addition, restrictions on partner attendance at ultrasound scans were advised by professional organisations [3,4]. However, inconsistent communication around guidelines, which were constantly updated in response to emerging (and often contradicting) knowledge about the virus and public health advice, resulted in confusion and ambiguity in how they were used [1]. Concerns were also raised about increasing variation in practice between clinical centres [5]. When UK lockdown restrictions began to ease, many partners were still unable to attend pregnancy scans due to reasons such as varying interpretation of guidelines, differing estates and local risk assessment. Parent advocacy groups considered the on-going restriction of partners at scans to be disproportionate in the response to COVID-19, calling for the guidance to be reviewed, and risks of virus transmission to be re-evaluated against the psychosocial risks in expectant parents [6]. The risk of psychological harm was especially concerning in parents whose scans demonstrated an unexpected physical fetal condition or fetal loss, as many pregnant women and people received this news alone [1]. Research exploring how the pandemic further impacted women who had experienced pregnancy loss suggests that feelings of grief, trauma and anxiety were exacerbated because of inadequate social support [7], thus highlighting the value of companionship during antenatal care.

### Parent-fetal bonding during COVID-19

The maternal prenatal bond is a complex entity thought to be influenced by various contextual and psychosocial factors including support [8], physical health [9] and the strength of the parental relationship [10]. Maternal anxiety during pregnancy is also considered to have a negative effect on the developing prenatal bond, albeit small [11]. Pregnancy is a transformational life event during which individuals must make significant changes to adjust to their new circumstances [12]. As a result, there is a high risk of new onset or recurrence of mental illness, including depression and anxiety [13]. The COVID-19 pandemic presented further stressors in addition to those already experienced by expectant parents, with studies reporting increasing levels of depression [14,15] and anxiety [16] in pregnant women compared to pre-pandemic levels. Prenatal maternal distress has been associated with impaired fetal neurological development and increased risk of mental health problems in later life [17]. It is also thought that anxiety during the pandemic may have been further increased in mothers of high-risk pregnancies [18], with a resultant negative impact on prenatal bonding demonstrated [19].

### Study rationale and aim

However, research studies exploring the effect of the COVID-19 pandemic on prenatal bonding have focussed predominantly on mothers and are generalised to consider the whole pregnancy experience, including labour and childbirth [20–22]. Whilst some do acknowledge antenatal ultrasound as part of the wider analysis, drawing more specific conclusions around the effect of ultrasound scanning during the pandemic on bonding is challenging because of the additional and external moderators. Focused research in this area is therefore warranted to gain a deeper understanding of how the changes to pregnancy imaging services have affected psychological and social domains of antenatal care [23]. The aim of this study was to gain insight from parents who had accessed pregnancy ultrasound scans during the COVID-19 pandemic, compare the experiences of mothers and fathers or partners, and to evaluate prenatal bonding during this time.

## Materials and methods

The study methods and results are reported as per the Checklist for Reporting Results of Internet E-Surveys (CHERRIES) [24]. Although a formal framework for reporting mixed methods research has yet to be published, the Checklist of Elements to Include in a Mixed Methods Manuscript has been used to guide the presentation of the methods and results [25].

An online, anonymous questionnaire was created using the secure Qualtrics XM^TM^ survey platform (www.qualtrics.com). This was reviewed by parent volunteers and representatives from UK-based antenatal support charity, Antenatal Results and Choices (ARC). In response to their feedback, minimal amendments were made to the phrasing of questions and overall survey structure to improve readability and usability. To improve overall completeness, the survey prompted participants to respond to all questions, however they could choose not to answer if preferred. Participants could also use navigation options within the Qualtrics XM^TM^ platform to move between questions and change their answers if desired. To ensure adequate time was given for all respondents to complete the survey, no restrictions for duration were enforced. For their convenience, participants also had the option to save their progress and return to complete the survey later. However, the “ballot-box stuffing” option was enabled in the platform to prevent multiple attempts at the survey.

The survey contained four sections. Part 1 contained questions regarding scan expectations or experiences. Part 2 included an adapted version of the Prenatal Attachment Inventory (PAI) to assess parent-fetal bonding, and the CORE-10 tool was used in part 3 to evaluate psychological distress. Participants were asked to provide basic demographic information (e.g. geographical location, ethnicity, education status) in part 4.

Circulation of the survey’s weblink to prospective participants was achieved through snowball sampling via social media platforms (e.g. Twitter, Facebook, LinkedIn) and word-of-mouth sharing. To be eligible for participation, the inclusion criteria required respondents to be an expectant or new parent aged 18 years or over, and either waiting for, or had attended a pregnancy ultrasound scan in the UK during the COVID-19 pandemic. The survey was open to respondents between 9^th^ March-25^th^ April 2021. During this 6-week data collection period, the UK was in its 3^rd^ national lockdown, which began on 6^th^ January 2021 [26].

A power calculation to determine sample size for this survey was based on estimates from studies using the maternal antenatal attachment scale [27] to compare change in bonding after ultrasound [28,29]. Using these studies to assume that prenatal bonding may be increased after fetal imaging by an approximate average of 3-points on the scale, an alpha of 0.05 and power of 80%, it was estimated that a minimum sample size of 39 participants was required in each scan group (e.g. waiting for scan vs. had scan) to avoid error in comparative analyses. A target sample size of 500 parent participants was set to absorb anticipated incomplete questionnaires, with the intention for this quota to be divided between the two groups, although this could not be controlled because of the sampling method.

### Parent expectations and experiences

This part of the survey captured parent expectations and experiences of pregnancy scans during the COVID-19 pandemic. Topics explored through these questions included searching for information about the scan, what might or did happen during the scan, and thoughts about the scan. A mixture of question types was used, with open-ended questions included to compliment the closed questions so that participants could further elaborate on their answers if they wanted to. Objective parental experience was quantitatively evaluated through closed-questions (e.g. “did you see images of the baby?”). Subjective parental experience was captured in the free-text responses which generated qualitative data. Participants were also asked to report their feelings of anxiety, excitement and satisfaction regarding their scan using a rating scale.

### Prenatal attachment inventory (PAI)

The original version of the prenatal attachment inventory (PAI) contains 21-items which measure the maternal-fetal relationship on a 4-point Likert scale from “Almost never” to “Almost always” [30]. As noted in a previous study [31], the PAI was modified for use by both parents in this survey by removing or rephrasing gendered items (e.g. “I try imaging what the baby is doing in there” becomes “I try to imagine what the baby does inside the womb”). The modified PAI contained 16-items and was reviewed by a group of maternal and paternal advisors to evaluate content validity. The response to each item in the PAI receives a score between 1–4, and these are combined to generate the total score. It is often considered that higher scores reflect a more developed bond, although it must be noted that no optimal score has been reported in the literature [32]. For the 16-item adapted PAI, the maximum possible score was 64.

It should also be noted that the relationship between expectant parents and their unborn babies is complex and definitions have evolved over time to reflect the growing body of research into this topic. Within the literature, studies refer to prenatal “attachment” and “bonding” interchangeably, however it has been suggested that the term attachment is less accurate as this implies a reciprocal relationship between the parent and the baby which is limited during the fetal period [33]. For simplicity, in this paper, the parent-fetal relationship is described as the prenatal bond throughout, although literature referring to attachment is also acknowledged and included.

### CORE-10

This generic measure of psychological distress contains 10-items related to well-being and general functioning. Participants are required to choose from one of five Likert responses (“Not at all,” “Only occasionally,” Sometimes,” “Often,” and “Most or all of the time”) which best reflects how often they have experienced symptoms in the past 7 days [34]. The highest distress response to each item scores 4-points, and the lowest scores 0-points. The maximum possible score is 40. The CORE-10 is validated for use in the perinatal population and commended for its brevity [35], as well as being simple to interpret with total scores less than 10 considered non-clinical at one end of the scale, and total scores of 25 or above corresponding with severe psychological distress at the other [36].

### Data analysis

A mixed methods approach was used for data analysis so that rich quantitative and qualitative insights could be extracted and developed from the data initially, and then combined to generate a more comprehensive perspective to address the research aim [25].

### Quantitative data analysis

The gendered terms “mother” and “father” are used throughout this paper as no non-binary parents completed part 4 of the questionnaire.

First, quantitative data were analysed in Microsoft Excel (version 2008, Microsoft Corporation, USA) and IBM SPSS Statistics (version 28, SPSS Inc, USA). In addition to descriptive statistics, parametric statistical analyses were performed as Q-Q plots demonstrated normally distributed data. Cronbach’s alpha was calculated to determine the reliability of the CORE-10 and adapted PAI tools in this study. Pearson and point bi-serial correlation coefficients were calculated to explore possible associations between variables [37]. Due to differences in group sizes, Welch’s t-test of unequal variances was used to compare means between parents who were waiting for a scan and those who already had been scanned, as well as between maternal and paternal participants. Further analyses of quantitative data from parents who had been scanned were performed to identify differences between maternal and paternal responses. As no fathers who were waiting for a scan took part in the survey, maternal vs. paternal comparisons could not be made for this group of parents. To identify any variations between the means of the post-scan PAI, CORE-10 and scan anxiety, excitement and satisfaction scores by timing of scan in relation to COVID-19 restrictions, analysis of variance (ANOVA) was used [37]. For this, parents were allocated to one of four groups which reflected the national COVID-19 restrictions depending on the timing of the scan [26]. Post-hoc testing was performed to further identify differences between groups that reached statistical significance on ANOVA. Statistical significance was determined using a value of p<0.05.

### Qualitative data analysis

Free-text responses to survey questions were collated and managed using NVivo qualitative data analysis software (version 12, QSR International). An inductive, thematic analysis was chosen to further explore parent experiences, primarily because it is well-suited to the study’s large and heterogeneous dataset. The flexibility of this approach also facilitated a thorough analysis of the qualitative responses because they could be coded at question or dataset level. The analysis sought to explore the research question ‘what was the parent experience of pregnancy ultrasound scans during the COVID-19 pandemic?’. After initial familiarisation with the dataset, the free-text survey responses were coded at the surface level for each individual question, and four core concepts around parent experiences of scans, partner attendance, parent-centred care and COVID-19 were generated. These codes were grouped into key concepts and combined with notes made during familiarisation to generate basic descriptive summaries which provided a general overview of the data. A second phase of coding was then undertaken on the whole dataset and provisional themes were developed and refined. During a final review of the data, the codes and provisional themes were then checked against the dataset for alignment and further refined as needed before being finalised as five core themes [38].

### Convergence of data

Key quantitative findings and qualitative codes were then recorded into a joint display matrix [39] and triangulated. Connections between the data were assessed to identify where findings could be confirmed or integrated with other findings to provide explanations in the case of contradictions. These were grouped around the core domains of: 1) anxiety; 2) excitement; 3) satisfaction; and 4) bonding, which were based on the structure of the survey questions. This process produced several integrated claims (IC) which could then be further developed or combined to generate a new claim if deeper understanding was required. To help provide a rich perspective on the full dataset, a final meta-inference, or “conclusion that connects or integrates various claims” [40] was developed using the integrated claims.

### Ethical considerations

Ethical approval for the study was received from the School of Health and Psychological Sciences at City, University of London (reference ETH2021-1240). Prior to accessing the survey, prospective participants were required to read the study information sheet which explained the purpose of the study, eligibility criteria and instructions for navigating the online platform. After reading the information, participants were then required to confirm their consent to take part in the study by marking a digital checkbox built into the survey platform [41]. Due to the potential for sensitive issues to be raised by the questionnaire, the psychological well-being of participants was fully considered in the study design. As all responses were anonymous, individual support could not be offered. However, a link to a collection of further online resources where participants could self-refer for UK-based perinatal mental health support was built into the Qualtrics XM^TM^ platform. Data management and research governance procedures were followed as per university guidance.

### Reflexivity statement

All authors are female and from a range of clinical academic backgrounds including medical imaging, midwifery and psychology. The first author is a registered diagnostic radiographer with specialist ultrasound training, and over 10 years’ experience of performing obstetric ultrasound examinations. During the COVID-19 pandemic, the first author was working in a full-time research role in a UK higher education institution. All authors believe that inclusive person-centred care is integral for the provision of pregnancy imaging, to facilitate a supportive experience for parents, birth partners and healthcare professionals alike. They also acknowledge the unique challenges raised by the pandemic, and that these impacted the lives of healthcare professionals as well as healthcare service provision.

### Public engagement

Seven parents (four mothers and three fathers) who had pregnancy ultrasound scans during the COVID-19 pandemic volunteered to review the preliminary findings and manuscript prior to journal submission. These parents responded to an invitation to review which was posted on social media. An infographic summary of the research was prepared and circulated to the parents, who were asked to comment on how well the findings reflected their personal experiences during this time. All parents providing feedback were entered into a prize draw for a gift voucher in recognition of their time and contributions. Their suggestions were collated and addressed in the final manuscript submission.

## Results

To minimise the potential for unforeseen psychological distress in responding to this survey, participants were free to answer the questions they felt comfortable with. Therefore, some responses are missing from the full dataset. All percentages have been calculated and reported to reflect the number of participants responding to the specific question, therefore these figures will vary.

### Participant characteristics

**Fig 1:**
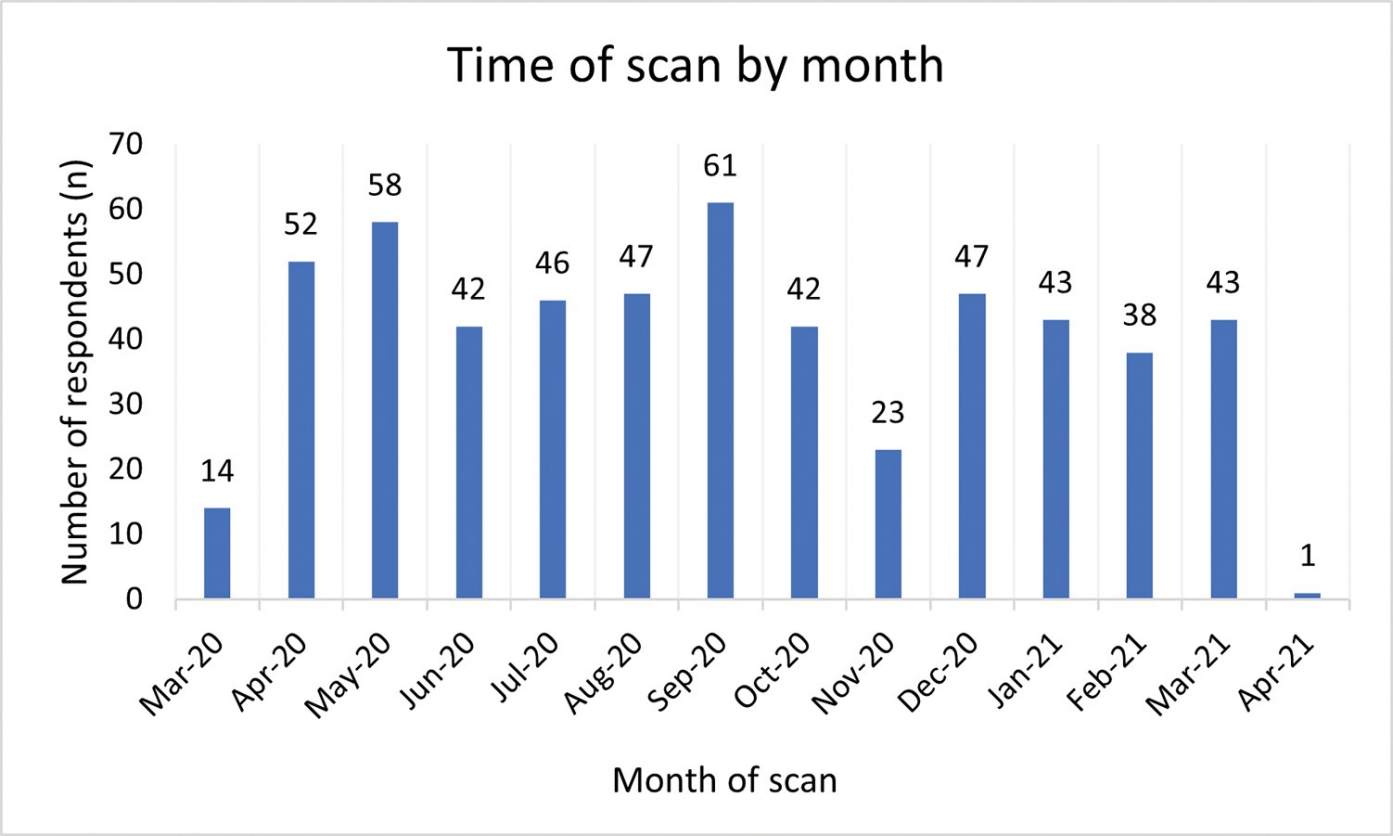
Timing of respondents’ pregnancy scan appointments during the pandemic.

The target sample size was exceeded, as 714 new and expectant parents consented to take part in the online survey. Of these, 96.4% (n = 688) reported they had attended a pregnancy ultrasound scan since March 2020 (Fig 1).

The remaining 3.6% (n = 26) were awaiting a scan at the time of completing the questionnaire. Across both groups of parents, the average completeness for the questionnaire was 79.8%. Most participants who answered the question (96.3%, n = 474) were the mother of the baby who was being, or had been scanned, and of white/British/Welsh/Scottish/Northern Irish/Gypsy or Irish Traveller ethnicity (94.1%, n = 461). In section four of the questionnaire, only 14 respondents answered that they were a father although it is believed that several others did not complete this part of the survey, which likely increases the actual total of fathers represented in the data. Most parents reported the setting for the scan was an NHS or public hospital (98.1%, n = 576). At the time of completing the survey, 47.4% of parents (n = 223) reported the pregnancy was on-going. For other parents, the baby had either been born (n = 220) or the pregnancy had ended (n = 28). Full participant characteristics are reported in Table 1.

**Table 1:** Participant characteristics.

		Waiting for scan	Had scan	TOTAL RESPONSES
	Consent to survey	26	688	714
	**Survey completeness**	75.04%	84.50%	Average = 79.77%
**Relationship to baby**	**Mother**	17	457	474
	**Father**	0	14†	14
	**Other**	0	4	4
**Previous scans**	**Yes**	17	556	573
	**No**	3	178	181
**Ethnicity**	**White**	13	448	461
	**Asian**	2	4	6
	**Black**	0	3	3
	**Mixed**	0	7	7
	**Prefer not to say**	1	1	2
	**Other**	1	10	11
**Education**	**Secondary**	1	28	29
	**College**	3	115	118
	**UG**	7	151	158
	**PG**	5	151	156
	**Doctorate**	0	20	20
	**Prefer not to say**	0	2	2
	**Other**	0	6	6
**Employment**	**Full time**	7	309	316
	**Part time**	4	108	112
	**Unemployed**	2	25	27
	**Student**	0	5	5
	**Other**	2	23	25
**Parental disclosure of physical health condition**	**Yes**	4	92	96
	**No**	12	371	383
**Parental disclosure of mental health condition**	**Yes**	6	165	171
	**No**	10	301	311
**Parental disclosure of currently prescribed medication**	**Yes**	5	136	141
	**No**	11	331	342

†Of these, n = 7 fathers attended the scan and n = 7 did not attend the scan.

Most parents reported scans during the first (46.9%, n = 271) and second (36.0%, n = 213) trimesters of pregnancy, which coincide with the routine fetal screening examinations offered in the UK as part of the NHS Fetal Anomaly Screening Programme between gestational ages of 11^+2^–14^+1^ weeks in the first trimester, and 18^+0^–20^+6^ weeks in the second trimester. For pregnant mothers who had been scanned (n = 565), more than half reported they had been alone during the examination (65.5%, n = 370). Most fathers and partners taking the survey (59.0%, n = 13) had not attended the scan. Eighty-five respondents (12.6%) described partner attendance at some routine scans and non-attendance at others. This seemed to vary between departments with some only allowing partner attendance at the first trimester scan (n = 12), and others only allowing partner attendance during the second trimester (n = 34). Non-routine examinations (e.g. scans in early pregnancy and third trimester) were most reported to have restrictions on partner attendance (n = 48) (Table 2).

**Table 2:** Respondents’ scan information.

		Waiting for scan	Had scan	TOTAL RESPONSES
**Geographical location for scan**	**North East**	0	15	15
	**North West**	3	96	99
	**West Midlands**	2	17	19
	**East Midlands**	3	76	79
	**Yorkshire**	3	15	18
	**East**	0	78	78
	**London**	1	57	58
	**South East**	4	59	63
	**South West**	3	127	130
	**Wales**	1	10	11
	**Scotland**	0	15	15
	**NI**	0	2	2
**Scan setting**	**NHS/public hospital**	19	557	576
	**Private**	1	10	11
**Timing of scan**	**Early pregnancy (≤10w)**	2	23	25
	**1**^**st**^ **Trimester**	6	265	271
	**2**^**nd**^ **Trimester**	3	210	213
	**3**^**rd**^ **Trimester**	9	73	82
**Timing of survey**	**Currently pregnant**		223	
	**No longer pregnant**		243	
	**Other**		4	

### Pregnancy ultrasound scanning during the COVID-19 pandemic

#### Searching for information

The majority of parents waiting for scan (60.0%, n = 12) reported that they had searched for information in advance of the scan, with the most frequently accessed source (75.0%, n = 9) being internet information (e.g. local hospital or NHS webpage). The most searched for information was related to who could attend the scan (91.6%, n = 11). In comparison, only 35.7% (n = 191) of parents who had been scanned stated they had searched for additional information after the examination. Again, the most frequently accessed post-scan resource was internet information (91.1%, n = 174). Following the scan, the most searched for information was regarding the results of the scan (84.2%, n = 160). There were 22 parents who indicated they had searched for other information related to fetal growth, placental position and unexpected physical conditions. Most parents (89.5%, n = 171) felt that they had found all the information they were looking for (Table 3).

**Table 3:** Parents’ self-reported information searching behaviours^†^.

		Waiting for scan (n, %)		Had scan (n, %)	
**1. Searched for information about the scan?**	**Yes**	12 (60.0%)		191 (35.7%)	
	**No**	8 (40.0%)		344 (64.3%)	
**2. Source of information searched?**	**Internet information page**	9 (75.0%)		174 (91.1%)	
	**Internet forum**	3 (25.0%)		88 (46.1%)	
	**Family/friends**	3 (25.0%)		84 (44.0%)	
	**Social media**	3 (25.0%)		61 (31.9%)	
	**Healthcare professional**	4 (30.0%)		55 (29.0%)	
	**Someone who attended the hospital**	2 (16.0%)		20 (10.5%)	
**3. Information searched?**		**Who can come to the scan**	11 (91.6%)	**Scan results**	160 (84.2%)
		**What the scan is for**	5 (41.6%)	**How baby looked on scan**	47 (24.6%)
		**What would happen during the scan**	3 (25.0%)	**What happened during the scan**	44 (23.0%)
		**What the baby will look like**	3 (25.0%)	**How I felt after the scan**	34 (17.8%)
		**Getting results**	3 (25.0%)		
		**How to prepare for the scan**	3 (25.0%)		
**4. Found all information required?**		Yes, fully	7 (58.3%)	Yes	171 (89.5%)
		Partially	5 (41.6%)	No	21 (11.0%)

^†^In Table 3, n denotes the number of participants selecting the question response. The total number of respondents to each of the questions in this table ranges between 12–20 for those waiting for scan, and 192–536 for those who had been scanned. For questions 2&3 in this table, participants could select multiple responses.

#### Expectations for the scan appointment

Most parents who were waiting for a scan expected that they would see images of their baby (85.0%, n = 17), that the sonographer would explain the images during the examination (70.0%, n = 14), and that they would be given a scan picture (65.0%, n = 13) (Table 4). The majority (73.7%, n = 14) stated they wanted a scan picture and reported that they would likely keep it as a memento for themselves (88.2%, n = 15).

**Table 4:** Expectations and experiences of the scan appointment^†^.

	Scan expectations–what will happen during the scan?	Scan experiences–what did happen during the scan?
	Waiting for scan (n, %)	Had scan (n, %)
**See images of the baby?**	17 (85.0%)	487 (97.0%)
**Get a picture?**	13 (65.0%)	493 (98.2%)
**See baby move?**	15 (75.0%)	451 (89.8%)
**Sonographer explains images?**	14 (70.0%)	433 (86.3%)
**Opportunity to ask questions?**	15 (75.0%)	369 (73.5%)

^†^In Table 4, n denotes the number of participants selecting the question response. The total number of respondents to each of the questions is 20 for those waiting for scan, and 502 for those who had been scanned.

#### Experiences of the scan appointment

For parents who had been scanned (n = 502), most saw images of the baby (97.0%, n = 487), saw the baby move (89.8%, n = 451) and had the scan images explained to them (86.3%, n = 433) (Table 4). A small proportion of respondents (2.0%, n = 10) reported that they had additional imaging performed, although no further details were provided about the type of imaging. Most parents (80.0%, n = 433) indicated they would be having, or did have other scans in the pregnancy, with several respondents to this question answering that they had either had, or were planning to book private scans (15.7%, n = 68) in addition to those offered by the NHS.

#### Pre and post scan anxiety, excitement and satisfaction

The mean anxiety score of parents waiting for scans was 6.40 (SD 2.62). For parents who had been scanned, the mean anxiety score was lower at 4.70 (SD 3.29). Mean excitement was 6.45 (SD 3.09) for parents awaiting scans and 7.25 (SD 2.67) for parents who had been scanned. No significant differences between pre- and post-scan anxiety, or pre and post-scan excitement were demonstrated (Table 5). No statistically significant correlation between pre-anxiety and pre-scan excitement levels was observed (Table 6).

**Table 5:** Pre and post scan comparisons of anxiety, excitement, bonding and psychological distress (Welch’s t-test).

	Waiting for scan	Had scan	Mean difference	t	Significance
**Anxiety** **(pre or post scan)**	6.40 (SD 2.62)	4.70 (SD 3.29)	-1.70	2.81	0.10
**Excitement** **(pre or post scan)**	6.45 (SD 3.09)	7.25 (SD 2.67)	+0.80	-1.14	0.27
**PAI** ^†^ **(pre or post scan)**	46.76 (SD 9.79)	46.77 (SD 8.16)	-0.01	0.00	1.00
**CORE-10** **(pre or post scan)**	10.88 (SD 5.96)	11.42 (SD 7.11)	-0.54	-0.36	0.72

^†^Only parents whose pregnancy was on-going at the time of taking part in this study were eligible to complete the PAI. There were 17 mothers waiting for scan and 235 parents who had been scanned and gave responses to the PAI.

**Table 6:** Pre and post-scan correlations between bonding (PAI), psychological distress (CORE-10) and feelings about the scan (anxiety, excitement, satisfaction and information searching).

Variables	Pearson (R)	R^2^	p-value^*†*^
**PAI and searching for information**	-0.05	0.00	0.44
**PAI and pre-scan anxiety**	0.24	0.06	0.36
**PAI and pre-scan excitement**	0.05	0.00	0.85
**PAI and post-scan anxiety**	0.05	0.00	0.42
**PAI and post-scan excitement**	0.25	0.06	<0.001**
**PAI and post-scan satisfaction**	0.09	0.01	0.167
**PAI and CORE-10**	-0.11	0.01	0.10
**CORE-10 and searching for information**	-0.25	0.06	<0.001**
**Pre-scan anxiety and searching for information**	-0.05	0.00	0.84
**Post-scan anxiety and searching for information**	-0.34	0.12	<0.001**
**Pre-scan anxiety and excitement**	-0.28	0.08	0.23
**Post-scan anxiety and excitement**	-0.36	0.13	<0.001**
**Post-scan anxiety and satisfaction**	-0.46	0.21	<0.001**
**Post-scan satisfaction and excitement**	0.49	0.24	<0.001**
**Post-scan satisfaction and searching for information**	0.22	0.05	<0.001**

^†^In Table 6, values marked with

** are significant at the level of p<0.05.

Negative (p<0.001) correlations were noted between post-scan anxiety and excitement (R = -0.36), and post-scan anxiety and satisfaction scores (R = -0.46). Parents who were more satisfied with their scan also scored more highly for excitement (R = 0.49, p<0.001). No association was demonstrated between pre-scan anxiety scores and searching for information, however, a higher post-scan anxiety score was correlated (p<0.001) with searching for information (R = -0.34).

Parents who had been scanned rated their overall satisfaction of the experience at an average of 6.46 (SD 2.75) (where 0 = not at all satisfied and 10 = extremely satisfied). A lower post-scan satisfaction score was associated (p<0.001) with information searching (R = 0.22).

### Reliability analysis

A reliability analysis using Cronbach’s alpha showed good internal consistency of the modified PAI (α = 0.885) and CORE-10 (α = 0.847).

### Pre and post-scan bonding (PAI) and psychological distress (CORE-10)

No significant difference in mean PAI score was seen between parents who were waiting for a scan (46.76, SD 9.79) and parents who had been scanned (46.77, SD 8.16). Pre-scan PAI score was not associated with pre-scan anxiety or excitement (Table 6). PAI score was positively correlated (p<0.001) with post-scan excitement (R = 0.25), although no association was demonstrated between PAI score and post-scan anxiety or satisfaction.

The average CORE-10 scores equate to mild-low level psychological distress within the group of respondents. No significant difference was seen between parents waiting for a scan and parents who had been scanned (Table 5). The mean CORE-10 score was higher (p<0.001) in parents who also reported a previous mental health condition compared to those with no history of mental health issues. For all parents, the total CORE-10 score was higher (p<0.001) in those who searched for more information about their scan (R = -0.25). No correlation between total PAI and CORE-10 score was demonstrated.

#### Comparison of maternal and paternal post-scan bonding and feelings about scan

All maternal participants reported they were the birthing parent, and all fathers reported they were the non-birthing parent, therefore no analyses of same-sex couples could be performed.

Post-scan bonding was significantly higher (p<0.05) in mothers (47.01, SD 7.97) compared to fathers (38.22, SD 10.73). Although paternal-fetal bonding was lower, there was no significant difference demonstrated between those who had attended scans and those who had not (Table 7). Partner attendance did not significantly affect maternal-fetal bonding either, with a mean PAI score in mothers whose partner had attended the scan of 47.60 (SD 7.41) compared to 45.72 (SD 8.87) of those whose partner had not.

**Table 7:** Comparison of maternal and paternal feelings following pregnancy scan (Welch’s t-test) ^†^.

	**Group maternal mean** **(n = 512)**	**Group paternal mean** **(n = 13)**	**Mean difference**	**t**	**Significance**
**Post scan anxiety**	3.67 (3.036)	3.67 (3.905)	0.00	0.00	0.99
**Post scan excitement**	7.48 (2.42)	6.22 (3.80)	1.25	0.98	0.35
**Post scan satisfaction**	7.16 (2.45)	6.67 (3.94)	0.49	0.37	0.72
**Post scan PAI**	47.01 (7.97)	38.22 (10.73)	8.79	2.43	0.04**
**Post scan CORE-10**	11.24 (6.51)	9.00 (6.35)	2.24	0.98	0.36
**Mothers only**	**Scanned with partner mean** **(n = 155)**	**Scanned alone mean** **(n = 305)**	**Mean difference**	**t**	**Significance**
**Post scan anxiety**	3.23 (2.86)	5.44 (3.25)	-2.21	-7.49	<0.001**
**Post scan excitement**	8.00 (2.06)	6.90 (2.83)	1.10	4.73	<0.001**
**Post scan satisfaction**	7.94 (1.84)	5.66 (2.82)	2.23	10.36	<0.001**
**Post scan PAI**	47.60 (7.41)	45.72 (8.87)	1.88	1.55	0.12
**Post scan CORE-10**	10.72 (6.63)	11.90 (7.43)	-1.19	-1.66	0.10
**Fathers only**	**At scan mean** **(n = 7)**	**Not at scan mean** **(n = 7)**	**Mean difference**	**t**	**Significance**
**Post scan anxiety**	2.86 (2.41)	7.14 (3.44)	-4.29	-2.70	0.02**
**Post scan excitement**	8.29 (1.11)	3.57 (3.05)	4.71	3.85	0.005**
**Post scan satisfaction**	8.43 (1.40)	3.00 (3.16)	5.43	4.15	0.003**
**Post scan PAI**	38.00 (9.92)	38.67 (14.64)	-0.67	-0.07	0.948
**Post scan CORE-10**	9.83 (8.06)	9.29 (5.88)	0.55	0.14	0.893

^†^In Table 7, n denotes the number of participants answering the question. Note that only parents whose pregnancy was on-going at the time of taking part in this study were eligible to complete the PAI–this was 226 mothers and 9 fathers. The scores are reported in the table as mean(standard deviation). Values marked with

** are significant at the level of p<0.05.

There was no significant difference (p = 0.36) in psychological distress (CORE-10) demonstrated between mothers and fathers. CORE-10 score was also not significantly affected for either parent by partner attendance at the scan.

When comparing parental mean scores for anxiety, excitement and satisfaction, no significant differences were noted between mothers and fathers generally. However, anxiety was significantly higher (p<0.05) in fathers who had not attended scans (7.14, SD 3.44) compared to those who had (2.86, SD 2.41). Paternal excitement and satisfaction was significantly higher (p<0.05) in those who had been present at the scan than those who had not. For mothers who had been scanned with their partners, there were significantly (p<0.001) lower levels of anxiety, and higher reported levels of excitement and satisfaction compared to those who had been scanned alone (Table 7).

Mean scores were also compared between parents who had prior experience of pregnancy ultrasound scans (either earlier in the current pregnancy or in a previous pregnancy) and those who had not. No significant differences were observed in post-scan anxiety, satisfaction, bonding or psychological distress (Table 8). However, parents with prior scan experience scored significantly higher (p<0.05) for excitement (7.67, SD 2.52) than those without (7.04, SD 2.72).

**Table 8:** Post-scan anxiety, excitement, satisfaction, bonding and psychological distress by parental experience of scans (Welch’s t-test) ^†^.

	No previous scan experience mean	Previous scan experience mean	Mean difference	t	Significance
**Anxiety**	4.67 (3.33)	4.78 (3.20)	0.111	0.367	0.714
**Excitement**	7.04 (2.72)	7.67 (2.52)	0.629	2.607	0.009**
**Satisfaction**	6.51 (2.75)	6.37 (2.74)	-0.137	-0.536	0.592
**PAI**	46.91 (8.26)	46.50 (8.39)	-0.414	-0.377	0.707
**CORE-10**	11.78 (7.35)	10.69 (6.50)	-1.092	-1.688	0.092

^†^In Table 8, the number of participants answering the question ranged from 170–172 for parents with previous scan experience and 318–353 without. Note that only parents whose pregnancy was on-going at the time of taking part in this study were eligible to complete the PAI–this was 90 with previous scan experience and 162 without. The scores are reported in the table as mean(standard deviation). Values marked with ** are significant at the level of p<0.05.

### Post-scan bonding and feelings about scan by timing of scan

ANOVA testing demonstrated that, in general, as the pandemic progressed, parental anxiety scores decreased while satisfaction scores increased (Table 9). However, two exceptions to this were observed. Firstly, there was no significant difference in anxiety score between parents scanned during November 2020 –February 2021 (3.73, SD 3.06) and March–May 2021 (3.26, SD 3.19). No significant difference in satisfaction was demonstrated between parents scanned during July–October 2020 (6.53, SD 2.81) and November 2020 –February 2021 (6.95, SD 2.50). Post-scan excitement, bonding and psychological distress scores did not significantly differ with the timing of the pregnancy ultrasound scan during the COVID-19 pandemic.

**Table 9:** Post-hoc analysis of post-scan anxiety and satisfaction by timing of scan during the COVID-19 pandemic (Welch’s t-test) ^†^.

	Scanned March 2020—June 2020 (1^st^ UK lockdown) (n = 159)	Scanned July 2020 –October 2020 (Local restrictions) (n = 167)	Scanned November 2020 -February 2021 (2^nd^/3^rd^ UK lockdown) (n = 153)	Scanned March 2021—May 2021 (Local restrictions) (n = 43)	Mean difference	t	Significance
**Anxiety**	5.70 (3.15)	5.00 (3.29)			0.7	1.97	0.05**
**Satisfaction**	5.52 (2.80)	6.53 (2.81)			-1.008	-3.23	<0.001**
**Anxiety**	5.70 (3.15)		3.73 (3.06)		1.975	5.622	<0.001**
**Satisfaction**	5.52 (2.80)		6.95 (2.50)		-1.428	-4.741	<0.001**
**Anxiety**	5.70 (3.15)			3.26 (3.19)	2.444	4.469	<0.001**
**Satisfaction**	5.52 (2.80)			7.95 (2.00)	-2.435	-6.447	<0.001**
**Anxiety**		5.00 (3.29)	3.73 (3.06)		1.275	3.598	<0.001**
**Satisfaction**		6.53 (2.81)	6.95 (2.50)		-0.420	-1.410	0.160
**Anxiety**		5.00 (3.29)		3.26 (3.19)	1.744	3.177	0.002**
**Satisfaction**		6.53 (2.81)		7.95 (2.00)	-1.426	-3.802	<0.001**
**Anxiety**			3.73 (3.06)	3.26 (3.19)	0.470	0.860	0.380
**Satisfaction**			6.95 (2.50)	7.95 (2.00)	-1.006	-2.748	0.007**

^†^In Table 9, n denotes the average number of participants answering the question. The scores are reported in the table as mean(standard deviation). Values marked with ** are significant at the level of p<0.05.

### Qualitative findings

**Fig 2:**
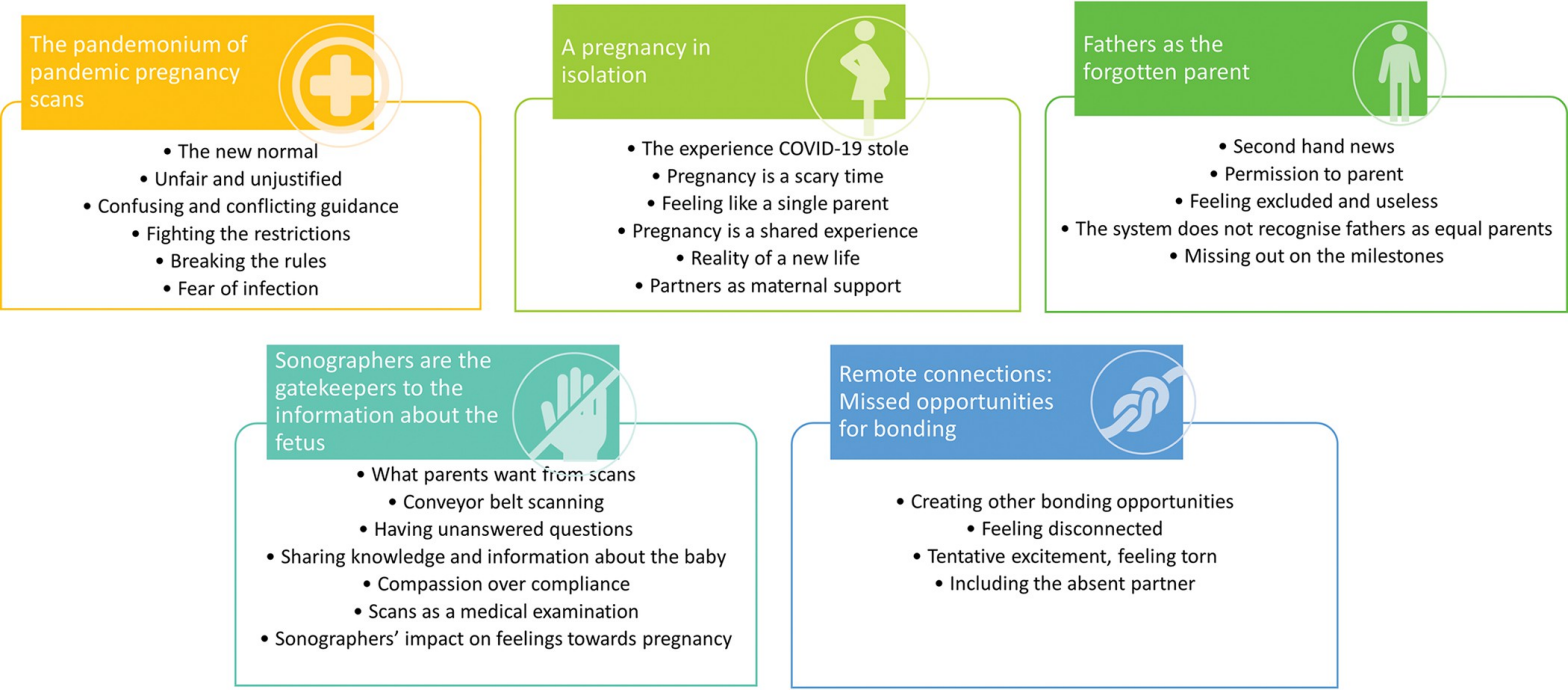
Key themes and codes.

Five core themes were developed in relation to parental experiences of pregnancy ultrasound scans during the pandemic: 1) the pandemonium of pandemic pregnancy scans; 2) fathers as the forgotten parent; 3) a pregnancy in isolation; 4) sonographers as the gatekeepers to the information about the fetus; and 5) remote connections: missed opportunities for bonding. These themes and their corresponding codes are presented in Fig 2. Illustrative quotations are used to underpin each theme’s description below.

### The pandemonium of pandemic pregnancy scans

This theme captured expectant parents’ perceptions of how the COVID-19 pandemic had impacted on their pregnancy scans, creating uncertainty and stress, specifically in relation to restrictions on partner attendance. Parents were understanding of the measures initially, but as lockdown restrictions began to ease around the country, the rationale for not reinstating partner attendance became a point of contention:

*“I understand why these are the way things are but think a priority for our society should be getting scans back to normal first instead of allowing pubs and restaurants and socialising…”*

Parents’ scepticism of the restrictions was further intensified by perceived inconsistencies in their enforcement, alongside other COVID-19 safety measures.

*“People were walking round the hospital with no masks including hospital staff. COVID rules were not adhered to at the hospital but yet my partner was not allowed to attend…”*

Examples of “double-standards” were often described, with parents receiving mixed messages from clinical departments about partner attendance. Some parents had received written confirmation that partners would be allowed into the scan room, only to be told on arrival at the hospital that this was not the case. This ambiguity raised questions of credibility, and parents rejected previous justifications for partner restrictions based on attempts to minimise virus transmission, claiming it was “based on rubbish.” Parents who were co-habiting found the restrictions particularly exasperating:

*“If you live in the same household, what difference does it make?!”*

Despite the guidance being issued nationally, this sense of unfairness was so profound that some parents felt compelled to actively contest the restrictions, seeking support for their efforts from legal and governmental sources. Challenging the right to have a partner attend the scan became a battle against the healthcare system with anger that was often projected onto the sonographers as they were the first point of in-person contact for parents. The overall perception of lack of transparency around the restrictions created contempt and frustration, and parents felt their best interests had not been fully considered.

*“Whilst I appreciate the need to keep staff safe, I really feel that the impact on parents due to the changes made to Maternity services as a whole and in particular ultrasound scans have not been thought through properly during the pandemic.”*

### Fathers as the forgotten parent

Measures introduced in response to COVID-19 served as a reflection of how partners are viewed by the healthcare system as an adjunct to pregnancy rather than an equal parent with their own rights.

*“…he was discriminated against for not being the one physically carrying the baby, despite it being just as much his child as mine.”*

Both mothers and fathers perceived that guidance unfairly favoured healthcare professionals’ needs over their own, criticising ill-thought-out actions that served to further emphasise how partners had been seen as a low priority.

*“I wasn’t allowed in due to COVID but a student was. Really couldn’t make it up.”*

Parents largely described partner attendance or non-attendance at scans by using terms such as “allowed” or “not allowed” which implied antipathy at the prospect of needing permission from the system in order to be a part of the experience. Non-attendance at scans led to fathers feeling excluded from the pregnancy experience and undermined as a parent. The feeling of “missing out” on both the scan event and what it symbolised in the on-going pregnancy was evident. Scans can be an opportunity for fathers to acquire new knowledge about the pregnancy and their baby at the same time as their pregnant partner, temporarily placing them into a privileged position they do not otherwise have access to. Having to learn about their unborn baby and the scan from their partner meant that fathers were completely reliant on their partner to relay details, which often failed to satisfy their individual needs for information:

*“I was still very nervous in case my wife missed telling me something.”*

Having restricted access to information about the pregnancy emphasised the disconnect experienced by many partners who could not attend the scan, and instead were required to wait outside of the hospital building. The feeling of being excluded, however, was not just limited to those who did not attend the scan. Partners also felt excluded within the scan room as they sat behind screens and were not acknowledged by sonographers.

*“…the person doing the scan did not include him / talk to him.”*

### A pregnancy in isolation

Due to changes in service provision, parents were required to adjust their expectations for scanning and as a result were resentful towards the pandemic for denying them the opportunity to share the scan experience with their partner. They described feeling as if something irreplaceable had been taken away, and how this had affected their overall attitude towards their pregnancy.

*“It was not having my partner there that made the whole experience completely different and not what I wanted, expected or ever [want] to go through again.”*

Fathers overwhelmingly described how they had wanted to attend the scan to provide support for their pregnant partner. In their first act of parenting, they saw their primary role as protector and advocate for their future family, wanting to take some of the responsibility of antenatal care from their partner:

*“It was a high-risk pregnancy, our first, and my wife needed support during scans and appointments which I could not give to her. There were times when the medical professionals were not listening to her and her needs, and I needed to be there advocate for her when she felt helpless and alone.”*

In attending their scans and other antenatal appointments alone, mothers saw themselves as single parents, highlighting the impact that being separated from their partners had on the perception of their family as a whole. This placed the onus of pregnancy exclusively on them, further exacerbating worries about potential complications and having to relay information to their partners.

*“Pregnant women … need the support of their partners during all scans and appointments. I have felt so anxious and stressed prior to and during scans that I have not been able to hear and process the important medical information provided.”*

Parents also spoke of their concerns regarding the potential impact that the additional stress of partner restrictions may have had on the pregnancy:

*“All the extra stress of having to go through this alone, scans and other appointments, isn’t good for mum or baby.”*

Anxiety surrounding the scan was described by almost all parents but was more evident in those who had experienced pregnancy-related complications and were being followed-up by clinical teams. Instead of alleviating maternal apprehension, scans often seemed to exacerbate it.

*“…I was actually made to feel like a nuisance and was brought to tears once I left as the experience was so awful.”*

### Sonographers as the gatekeepers to the information about the fetus

Parents often attended for their appointment with the fundamental expectation that the scan would provide a chance to receive additional knowledge about their baby. The level of information about the baby that parents could access was perceived to be governed by the sonographer performing the scan. When sonographers openly shared information about the baby, parents described a more positive experience.

*“The lady doing it made me feel at ease. She would let me know what she was doing. She’d show me the baby and what he [the baby] was up to…pointing out body parts.”*

However, this was not the case for all parents, and some felt as if they had been actively kept in the dark about what had happened during the scan and the results. For mothers particularly, this placed them in an uncomfortably passive role during the scan process, where the scan was largely done to them without their involvement and resulted in a lack of knowledge afterwards.

*“I couldn’t see the screen to know what was going on. Most of the scan was done in silence and I was handed the photographs at the end without even being sure everything was okay.”*

Many of those who felt uninformed following the scan later went on to search for further information, particularly after additional or unexpected findings were identified and not fully discussed, which often did not provide the reassurance that had been hoped for:

*“I wasn’t told what it would mean [my daughter having a small head] so ended up Googling it and ended up increasing my anxiety.”*

When sonographers narrated and explained the scan appearances, parents felt more included in the process and felt that they had received a higher quality scan and a more personal care experience. Conversely, the concept of “conveyor belt scanning” was alluded to by the parents who had been given little in the way of information about the scan and their baby and were left with the impression that they had been a waste of the sonographers’ time. This impacted negatively on parents’ overall perception of the scan experience and feelings towards their pregnancy:

*“She [the sonographer] completely took away any excitement I could have felt because of the way she was.”*

Not all parents shared this view, however, highlighting the need to improve parental awareness of the medical rationale behind pregnancy ultrasound examinations, and manage expectations around the more social elements of scanning.

*“Whilst it’s nice for the sonographer to explain and take questions and have a good bedside manner, all that really needs to be done is the health screening and it would help if more women realised this.”*

### Remote connections: Missed opportunities for bonding

Pregnancy scans were generally considered to be a positive event, whereby expectant parents could see and connect with their unborn babies in “real-time”. Although most parents were given pictures from the scan as a memento, this was not seen as an adequate substitute for the live experience. For partners who could not attend the scan, this was perceived as having had a considerable impact on their developing bond with the baby and how they then processed the reality of the pregnancy.

*“He felt disconnected from the pregnancy and not as involved as he could be.”*

This disengagement of partners also affected mothers, who described feelings of guilt for having enjoyed the scan and time with their unborn baby in the absence of their partner. To mitigate this personal conflict, they would purposely downplay their scan experience when relaying it back to their partners:

*“I didn’t want to appear over excited (even though I was) as I could see the heartbreak in his face missing out on such a special moment for us…”*

Many parents described how they created their own opportunities for bonding by booking non-medical scans at private clinics, although this was recognised as a privilege.

*“We were lucky that we were in a position to be able to pay for a private scan but not everyone can.”*

During these scans parents could experience three and four-dimensional imaging of their baby, something which is not routinely offered by the public health services. Parents felt these scans would enable them to “properly see” their babies in a way that they had not been able to in hospital departments. At later gestations, the timing of these was carefully considered to complement the clinical scans and “check-in with baby”. However, in light of the partner restrictions many parents also chose to be scanned in very early pregnancy to avoid the possibility of receiving unexpected news without partner support:

*“I absolutely couldn’t face finding out if the pregnancy had failed on my own which was my concern.”*

Parents commented on the use of digital alternatives such as video-calling or taking short recordings of the scan that could be shared with partners to overcome the challenge of not being physically present. However, parents reported inconsistencies in this practice, which created a further source of confusion for parents alongside partner restrictions, and was considered to be unjust:

*“I felt I could have easily called my partner or taken a video so that he could have been part of such a special moment. I can’t see why this would have been a problem which was the most frustrating thing.”*

### Integration of quantitative and qualitative data

**Fig 3:**
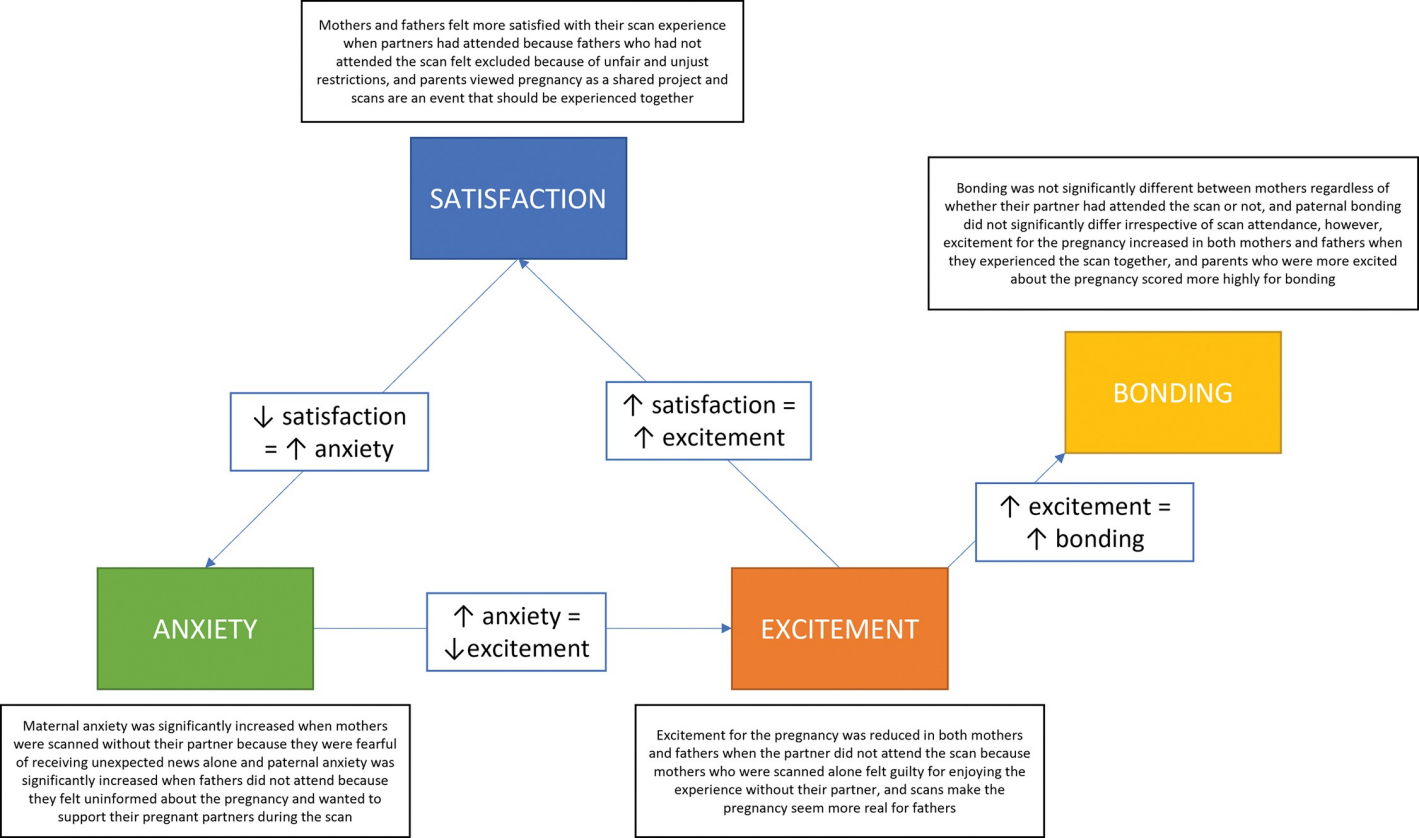
Visual representation of integrated claims developed for each domain.

Integration of key quantitative and qualitative data through the joint display matrix facilitated triangulation to offer insight and further clarity on the findings. These were largely explanatory and centred around partner attendance at scans during the COVID-19 pandemic (Table 10). The integrated claims were further explored using other claims generated through the matrix to try to provide a comprehensive, overarching meta-inference. The interwoven nature of the survey domains, quantitative claims and qualitative codes is demonstrated in Fig 3.

**Table 10:** Display matrix for development of the meta-inference.

Domain	Quantitative claim	Qualitative code(s)	Integrated claims	Connection between quantitative claim and qualitative data	Illustrative quotation
Anxiety	Maternal anxiety was significantly increased when mothers were scanned without their partner	Feeling like a single parent Scans as a medical examination	[IC1] Maternal anxiety was significantly increased when mothers were scanned without their partner **because** mothers were fearful of receiving unexpected news alone	Explanation	*“I was extremely anxious about anything being wrong and being on my own with my husband not allowed to attend”*
	Paternal anxiety was significantly increased when fathers did not attend the scan	Partners as maternal support Second hand news	[IC2] Paternal anxiety was significantly increased when they did not attend the scan **because** they felt uninformed about the pregnancy **and** wanted to support their pregnant partners during the scan	Explanation	*“My wife needed support during scans […] which I could not give to her […] I needed to be there to advocate for her when she felt helpless and alone”*
Excitement	Excitement for the pregnancy was reduced in both mothers and fathers when the partner did not attend the scan	The experience COVID-19 stole Missing out on the milestones Reality of a new life	[IC3] Excitement for the pregnancy was reduced in both mothers and fathers when the partner did not attend the scan **because** mothers who were scanned alone felt guilty for enjoying the experience without their partner, **and** scans make the pregnancy seem more real for fathers	Explanation	*“I didn’t want to appear over excited (even though I was) as I could see the heartbreak in his face missing out on such a special moment for us”*
Satisfaction	Mothers and fathers felt more satisfied with their scan experience when partners had attended	Feeling excluded and useless Unfair and unjustified Pregnancy is a shared experience What parents want from scans	[IC4] Mothers and fathers felt more satisfied with their scan experience when partners had attended **because** fathers who had not attended the scan felt excluded **because** of unfair and unjustified restrictions **and** parents viewed pregnancy as a shared project and scans are an event that should be experienced together	Explanation	*“It led to anxiety from not being present rather than the joy of seeing our baby”*
Bonding	Bonding was not significantly changed between mothers waiting for scans and those who had been scanned	Tentative excitement, feeling torn Scans as a medical examination	[IC5]† Bonding was not significantly changed between mothers waiting for scans and those who had been scans **because** after the scan, mothers maintain some emotional distance from the baby in case of an unexpected outcome	Explanation	*“Still have a long way to go until a safe delivery of a baby”*
Bonding	Bonding was not significantly different between mothers regardless of whether their partner had attended the scan or not	Feeling disconnected	[IC6]† Bonding was not significantly different between mothers whose partners attended the scan and those whose partner did not **but** mothers whose partners did not attend the scan perceived their emotional connection to the baby was reduced	Contradiction	*“I just felt I was going through the motions and couldn’t be excited as my partner had missed out”*
Bonding	Paternal bonding did not significantly differ irrespective of scan attendance	The system does not recognise fathers as equal parents Missing out on the milestones	[IC7]† Paternal bonding did not significantly differ irrespective of scan attendance, **but** mothers perceived that not attending the scan had been detrimental to their partner’s bonding	Contradiction	*“One of the only ways he could bond with the baby during pregnancy had been taken away from him”*
Bonding	Bonding was not significantly different between mothers regardless of whether their partner had attended the scan or not	Scan as a medical examination Sharing knowledge and information about the baby	[IC8] Bonding was not significantly different between mothers regardless of whether their partner had attended the scan or not, **and** paternal bonding did not significantly differ irrespective of scan attendance, **however** excitement for the pregnancy increased in both mothers and fathers when they experienced the scan together, **and** parents who were more excited about the scan scored more highly for bonding	Juxtaposition	*“Sharing the experience was amazing*, *got us even more excited about the baby”*
Bonding	Paternal bonding did not significantly differ irrespective of scan attendance	Partners as maternal support			
Excitement	Excitement for the pregnancy increased in both mothers and fathers when they experienced the scan together	Pregnancy is a shared experience			
Excitement	Parents who were more excited about the scan scored more highly for bonding	Reality of a new life			
**Integrated Claims**			**Meta-inference**		**Illustrative quotation**
[IC1] Maternal anxiety was significantly increased when mothers were scanned without their partner because mothers were fearful of receiving unexpected news alone			Maternal anxiety was significantly increased when mothers were scanned without their partner because they were fearful of receiving unexpected news alone. Paternal anxiety was significantly increased when they did not attend the scan because they felt uninformed about the pregnancy and wanted to support their pregnant partners during the scan. Excitement for the pregnancy was reduced in both mothers and fathers when the partner did not attend the scan. **This is because** mothers who were scanned alone felt guilty for enjoying the experience without their partner, and scans make the pregnancy seem more real for fathers. **In addition,** mothers and fathers felt more satisfied with their scan experience when partners had attended **because** fathers who had not attended the scan felt excluded and parents viewed scans as a pregnancy-related event that should be experienced together. **Although** bonding was not significantly different between mothers regardless of whether their partner had attended the scan or not, **and** paternal bonding did not significantly differ irrespective of scan attendance, excitement for the pregnancy increased in both mothers and fathers when they experienced the scan together, **and** parents who were more excited about the pregnancy scored more highly for bonding.		*“Having a partner there would have completely changed the experience”*
[IC2] Paternal anxiety was significantly increased when they did not attend the scan because they felt uninformed about the pregnancy and wanted to support their pregnant partners during the scan					
[IC3] Excitement for the pregnancy was reduced in both mothers and fathers when the partner did not attend the scan because mothers who were scanned alone felt guilty for enjoying the experience without their partner, and scans make the pregnancy seem more real for fathers					
[IC4] Mothers and fathers felt more satisfied with their scan experience when partners had attended because fathers who had not attended the scan felt excluded and parents viewed pregnancy as a shared project and scans are an event that should be experienced together					
[IC8] Bonding was not significantly different between mothers regardless of whether their partner had attended the scan or not, **and** paternal bonding did not significantly differ irrespective of scan attendance, **however** excitement for the pregnancy increased in both mothers and fathers when they experienced the scan together, **and** parents who were more excited about the pregnancy scored more highly for bonding					

†In table 8, integrated claims 5–7 have been further developed to generate integrated claim 8 for use in the meta-inference.

The following meta-inference was developed from the study data:

During the COVID-19 pandemic, maternal anxiety was significantly increased when mothers were scanned without their partner because they were fearful of receiving unexpected news alone. Paternal anxiety was significantly increased when they did not attend the scan because they felt uninformed about the pregnancy and wanted to support their pregnant partners during the scan. Excitement for the pregnancy was reduced in both mothers and fathers when the partner did not attend the scan. This is because mothers who were scanned alone felt guilty for enjoying the experience without their partner, and scans make the pregnancy seem more real for fathers. In addition, mothers and fathers felt more satisfied with their scan experience when partners had attended because those who had not attended the scan felt excluded and parents viewed scans as a pregnancy-related event that should be experienced together. Although bonding was not significantly different between mothers regardless of whether their partner had attended the scan or not, and paternal bonding did not significantly differ irrespective of scan attendance, excitement for the pregnancy increased in both mothers and fathers when they experienced the scan together, and parents who were more excited about the pregnancy scored more highly for bonding.

### Discussion

The findings from this study demonstrate how pandemic-related changes with regards to partner attendance at pregnancy ultrasound scans created further anxiety for partners in addition to their general concerns around fetal health and wellbeing. This had a significant effect on scan satisfaction and overall excitement for the pregnancy. For mothers, this also resulted in a perceived negative effect on their emotional connection to their unborn baby. Partner attendance at the scan was highlighted in four of the five key themes developed from the survey responses. Many parents also commented how this was a central factor in determining the scores they gave for anxiety, excitement and satisfaction. In keeping with these findings, Schaal et al reported that the greatest worry for pregnant women during the pandemic, was that their partners would not be present during birth or that they would not be visited whilst in hospital [21]. This highlights the importance of partners for maternal support and companionship throughout the pregnancy.

### COVID-19 effect on prenatal bonding

Several tools to assess prenatal bonding are used in research literature, although the most common are variations of the PAI, Maternal Antenatal Attachment Scale (MAAS) and Maternal-Fetal Attachment Scale (MFAS) [27,30,42]. Despite these objective measures, determining an optimal bonding score is challenging because whilst a score may be statistically significant within an analysis, this may not represent clinical significance [32]. Previous studies using the MAAS define a threshold of 80% of the global score to differentiate between low and high bonding [9,20]. Using this definition, the average scores of parents completing the modified PAI in this study would be classified as low bonding. However, these findings are more comparable with those of Albayrak *et al* who reported mean bonding scores using the Turkish version of the PAI of 75.8% and 70.8% in mothers with low and high anxiety and obsession around COVID-19 respectively [22].

As no significant difference in bonding was demonstrated between mothers in this study regardless of scan status (e.g. waiting for scan, scanned alone or scanned with partner), this may suggest that any interpretation of low bonding in this sample is more likely to be related to the wider impact of the pandemic on levels of maternal anxiety [22] rather than directly attributable to the changes to the provision of pregnancy ultrasound scans performed during this time. Anxiety during pregnancy has been previously associated with decreased prenatal bonding [8]. This is thought to be because mothers who are preoccupied with other stressors may be distracted from thinking about their pregnancy, resulting in a decrease in emotional connection towards their unborn baby [43]. This explanation may reflect findings from the thematic analysis of the free-text parental responses which described mothers’ feelings of reduced bonding, even though the PAI scores were unaffected. Alternatively, some parents may not have been comfortable to reveal their true feelings in this survey, and therefore may have modified their responses to the PAI. The reluctance to disclose information that could leave parents feeling vulnerable to negative judgement by others (including healthcare professionals) is not uncommon in the perinatal setting, and has been identified as a barrier to parents seeking further support [44].

In this study, bonding was significantly lower in fathers and partners than in mothers. However, no significant difference in bonding score was demonstrated between those who had attended the scan and those who had not. This implies that amongst this survey’s respondents, the scans did not influence bonding. The finding of lower paternal and partner bonding is consistent with other studies that report lower levels of bonding compared to mothers [27,45,46]. The development of the prenatal bond is thought to be an ongoing process which intensifies during pregnancy as parents engage more on an emotional level with their unborn baby. For this reason, it could be hypothesised that the maternal prenatal bond is accelerated as a result of their privileged embodied knowledge of the pregnancy [47]. In relation to scanning, it has been suggested that changes to paternal bonding may be dependent on the timing of the scan during the pregnancy [28], with earlier scans which confirm the viability of the pregnancy and the subsequent reality of impending parenthood, appearing to be more influential [10]. Fathers’ response to pregnancy ultrasound is thought to be a predictor for prenatal bonding [10], and this has potentially significant implications considering the further association between paternal support, maternal bonding and postnatal attachment [48].

### COVID-19 effect on scan experiences

Many parents described a sense of loss for their imagined pregnancy scan experience which had been taken away by COVID-19. This finding was also evident in recent studies evaluating the wider pregnancy and birthing experience and reflects how parents have felt their expectations for care have not been met [49–51]. Managing parental expectations of imaging in pregnancy became more challenging as sonographers attempted to balance parent-centred care and the social restrictions imposed by COVID-19. Pregnancy is generally considered to be a social event [52] and scans provide an opportunity for parents and their wider support networks to “meet” and get to know the baby before birth [53]. The prospect of a personalised care experience that can be shared and enjoyed with others is often how private providers promote their scan packages [54], which can include additional extras not offered during clinical examinations such as 4-dimensional imaging, high-quality prints and recordings of the fetal heartbeat.

In this survey, 48 parents mentioned they had booked or were planning to book a private scan in addition to those offered as part of their antenatal care pathway. Nearly all explained that this was so both parents could experience the scan together. Sharing the scan experience was important to parents for two reasons; firstly, for support in the event of unexpected news, and secondly for fathers and partners to feel involved with the pregnancy. As fathers lack embodied knowledge of the pregnancy, scans provide an opportunity for both parents to acquire new insights about their unborn baby simultaneously [47]. Sharing the scan experience can also create a sense of “togetherness” which helps to provide pregnant people with security and reassurance of their partner’s investment in, and commitment to the pregnancy and postnatal emotional and practical support [55]. Goyal *et al* report that mothers felt detached from their partners when they had not attended antenatal appointments [51]. A key finding from this survey was how mothers perceived the absence of their partner at the scan to have had a negative effect on bonding. Although this was not confirmed by the PAI scores which remained unchanged, this finding has been previously acknowledged by Göbel *et al*, who reported that increased maternal anxiety may lead to mothers’ perception of reduced emotional proximity to their baby [43]. It may also be suggested that mothers were also concerned about the possible negative effect of the restrictions on their relationship with their partner [55], which may have further influenced their feelings towards their baby. Our findings demonstrated that parents who were scanned at a later timepoint during the COVID-19 pandemic had significantly lower anxiety. This may be explained by a combination of increased information and understanding of the pandemic over time, as well as the removal of restrictions on partner attendance at scans towards the end of 2020 [56].

### The role of the sonographer during the COVID-19 pandemic

Despite their frustration about the restrictions, parents recognised the distress that adhering to guidance around partner attendance at scans had caused for sonographers, particularly where unexpected or difficult findings had been identified during the examination. A sub-theme of “compassion over compliance” was developed from the survey responses to capture parents’ reflections of how sonographers had demonstrated empathy for those whose scan experiences had been affected by the restrictions by giving printed scan photos at no charge and “sneaking” partners into the scan room to be given unexpected news as a couple. Other studies have reported that parents appreciated when healthcare professionals validated their feelings of disappointment with the situation [51], and used technology to facilitate inclusion of partners from outside of the scan room [50]. Whilst this was no substitute for having been physically present, it was considered better than missing out completely and so when video-calling options were not available, this became a further source of anxiety and stress in parents who felt like they were being given inconsistent and ambiguous guidance [57].

### Pregnancy companionship

“Fathers as the forgotten parent” was a key theme developed, and further supports the previously acknowledged lack of a family-centric approach to antenatal care [58]. During the pandemic, changes were made to care provision which prioritised infection control above psychological stress, and ultimately conceptualised partners as visitors rather than as parents and birth companions [52]. The concept of companionship in antenatal care is often described in relation to labour and childbirth, and it is considered by the World Health Organisation as integral to facilitating a positive parental experience [59]. Companionship may be provided by fathers and partners, family members, friends and healthcare professionals who give information support, advocacy, practical support and emotional support to pregnant women and people [60]. The benefits of companionship on infant outcomes and parental mental health are also acknowledged; associations between maternal health behaviours in pregnancy (e.g. cessation of smoking and consumption of alcohol) [61] and improved pre and postnatal bonding [62] have been reported when mothers feel adequately supported during pregnancy.

In restricting partner attendances during the COVID-19 pandemic, partners not only missed out on seeing their unborn baby during the ultrasound scan, but the potential to access to mental health services. For example, opportunities for sonographers to check-in with partners’ well-being and facilitate support interventions in a timely manner may have been lost, and this has been shown previously within the wider antenatal care pathway to have serious negative implications on maternal and fetal/infant outcomes [63]. The importance of adopting a parent-centred approach to care has also been previously identified as essential to a satisfactory scan experience, with the role of the sonographer considered integral to co-constructing parental knowledge and understanding about their unborn baby through their interpretation and narration of ultrasound images [53]. In this study, parental satisfaction increased as the COVID-19 pandemic progressed and lockdown restrictions were lifted. Satisfaction of experience may be a significant moderator for perinatal mental health, indeed, dissatisfaction with the birth process from inadequate partner support has been previously associated with postpartum depression [64].

### Strengths and limitations

As this was a UK-wide survey of parents, the responses provided are not limited to a single healthcare facility. In addition, the CORE-10 and modified PAI tools used demonstrated high reliability with Cronbach’s alpha. The convenience of the online and anonymous survey made it easier for parents to express their thoughts freely, which increases confidence that the findings are reflective of the experiences during this time. Separate quantitative and qualitative analyses not only produced rich findings, but new insights were generated because of the integration process [39]. This process also demonstrated trustworthiness as the quantitative and qualitative findings were largely complimentary.

A limitation associated with cross-sectional surveys is that they only capture a single moment in time, and results can be exaggerated by extreme responses from those who are more motivated to take part [65]. In addition, despite recruitment flyers explicitly stating that all parents were eligible to complete the survey, the proportion of parents waiting for scans and the number of fathers and partners was low. No non-binary parents took part in this study. When the survey was live, the intention was to have comparable numbers of respondents within the parent groups, however the final totals were skewed towards mothers who had been scanned. This could make statistical interpretation or generalisation more challenging as the results are not as powerful as if the optimal sample sizes had been achieved. Low uptake of fathers and partners is not uncommon in antenatal research and indeed antenatal care more holistically. Partners are often underrepresented, perceiving that, as they are not pregnant themselves, their perspectives are not relevant [66]. Similarly, there was limited variation in the ethnicity of parents completing this survey. At the time the survey was live, many other researchers were utilising online questionnaires whilst face-to-face data collection methods were restricted. This could have led to some prospective participants feeling over-researched and thus deciding not to take part in this study. Homogeneity within the population may also have occurred if the snowball sampling did not effectively reach underrepresented groups of parents. The lack of diversity in COVID-19 related antenatal research has been acknowledged [20,51] and highlights the need for more inclusive practices in research design and recruitment to gain a deeper understanding of all parents’ experiences during this time. Furthermore, other information could be collected in future studies to provide deeper insight and explanation to the findings. For example, postnatal data may be useful to explore potential associations between parental anxiety and pregnancy outcome. In addition, this survey did not ask participants to provide in-depth information about their obstetric history or personal life, which may be important factors to consider when interpreting data around parental anxiety [67].

### Recommendations for practice

This study has highlighted the immediate effect of the restrictions on partner attendance at scans during the pandemic on parent experiences of antenatal imaging and prenatal bonding, although the longer-term implications for parents and their infants may not be fully understood for several years. However, some recommendations for future practice can be developed from the literature.

1. Unexpected changes to the pregnancy and birthing experience because of COVID-19 have been associated with symptoms of anxiety and post-traumatic stress disorder [68], therefore parents who experienced antenatal care during the pandemic may benefit from additional follow-up and mental health interventions in the post-pandemic era. Opportunities for parents to access perinatal mental health services could be extended and made more inclusive of partners [69], and specialist training for healthcare professionals by psychological therapy teams may facilitate screening during pregnancy to improve early identification of parents in need of support [70]. In their parent-facing role, sonographers may be ideally positioned to recognise parents experiencing mental health difficulties and introduce resources, however this additional responsibility must be carefully balanced alongside existing clinical duties so as not to further increase workload and job demands within the profession [71].
2. Similarly, initiatives to promote staff wellbeing during the pandemic should be continued to alleviate burnout [72] and help to mitigate high sonographer attrition from obstetric services specifically of the NHS workforce in general in response to the pandemic [71]. As sonographers are central to parental experiences of pregnancy scans, promoting, offering training about, and practicing parent-centred care that is inclusive of fathers and partners as birth companions rather than visitors [52] may also contribute to improved satisfaction and perception of care, and enhanced prenatal bonding in the future.
3. More formalised, publicly available and versatile (in terms of content and format) information about what service users could expect from antenatal scans might be useful [73], as some parents in this study indicated they still had unanswered questions despite searching for additional information either prior to the scan, or after the appointment. Sonographers could be key in working with parents to co-develop that resource. Enhanced training for sonographers on key concepts and practices of parent-centred care would be vital to ensure they are equipped and empowered to address service user queries and manage expectations.

### Conclusion

Restrictions on partner attendance at scans were introduced with the intention of minimising virus transmission during the COVID-19 pandemic. Significant differences in parental feelings of anxiety, excitement and satisfaction between parents were correlated with partner attendance at the scan. Partner attendance was important to parent satisfaction, which was increased when both parents were present at the scan. When partners did not attend the scan, parental anxiety was higher, thus parents who had pregnancy scans during the COVID-19 pandemic may benefit from additional mental health support in early parenthood. Although no demonstrable change in prenatal bonding because of the restrictions was recorded in this study, the findings of this UK-wide survey demonstrated that bonding was lower in fathers compared to mothers. Data triangulation suggested maternal guilt in enjoying the scan without their partner, and paternal frustration at being excluded from the scan and being unable to provide support to their pregnant partner. Parental feelings of excitement about the pregnancy were positively correlated with increased prenatal bonding, highlighting the power of antenatal ultrasound scans as an opportunity for expectant parents to engage with their unborn babies, and the integral role of sonographers in providing individualised, parent-centred care to support this.
